# The Role of Alcohol in Initial Help-Seeking Telephone Calls About Domestic Violence to the Police

**DOI:** 10.1177/10778012241259725

**Published:** 2024-08-09

**Authors:** Emma Richardson, Marc Alexander, Elizabeth Stokoe

**Affiliations:** 1 Department of Communication and Media, School of Social Science and Humanities, 5156 Loughborough University, Loughborough, UK; 2 School of Social Sciences, 3120 Heriot-Watt University, Edinburgh, UK; 3 Department of Psychological and Behavioural Science, The London School of Economics and Political Science, London, UK

**Keywords:** domestic violence, emergency calls, alcohol consumption, question design, conversation analysis

## Abstract

This article investigates how domestic violence and abuse (DVA), its underreporting and its links with alcohol consumption, manifest in and impact the outcome of help-seeking telephone calls to U.K.-based police services. Conversation analysis of call-takers’ questions about alcohol found that they either (a) focused only on the perpetrator's drinking, and occurred after informing callers that help was being dispatched, or (b) targeted both victims’ and perpetrators’ drinking and complicated the decisions to dispatch police assistance. The article helps specify the communicative practices that may constitute victims’ negative experiences of disclosing DVA to the police.

## Introduction

Domestic violence is a prevalent, yet underreported, gendered crime. The link between a male partner's alcohol consumption and the perpetration of domestic abuse against a female partner has been consistently evidenced (see Foran & O’Leary, 2008; Leonard & Quigley, 2017). Furthermore, a report entitled *The Nature of Violent Crime in England and Wales* revealed that victims of domestic violence believed that perpetrator(s) were under the influence of alcohol in 34% of cases (Office for National Statistics, 2020). This article explores the interrelationships between the gendered social problems of aggression, alcohol consumption, and domestic violence and abuse (henceforth DVA), and the (under)reporting of the latter. We consider how these problems may manifest in initial reports of DVA in telephone calls to police services. We focus particularly on the communicative practices that comprise these calls, and specifically on how call-takers’ questions about alcohol consumption might be a contributing factor to the negative experiences callers report when disclosing DVA to the police.

We start by summarizing what we know about the gendered nature of DVA, its links with alcohol, and the barriers to its reporting. There is a breadth of international, interdisciplinary literature examining these topics, spanning decades (Devaney et al., 2021). However, what we know less about, and what this article investigates, is how such issues interconnect victims, perpetrators, and institutions themselves during initial reports of DVA to police emergency services. By analyzing reports as they happen, rather than via post hoc accounts thereof, the article provides direct access to victims’ voices in a setting where the stakes are immediate, live, and high.

### Domestic Violence and Abuse, Alcohol, and Barriers to Formal Reporting

DVA is a prevalent, gendered crime; in the United Kingdom, the latest figures at the time of writing indicate that seven in 100 women and three in 100 men were victims of DVA in 2021 (Office for National Statistics, 2022). While victims of DVA include men (e.g., Dixon et al., 2020), women are victimized by all types of violence and abuse at a higher rate, although men are more likely to be stalked (Office for National Statistics, 2022).

The links between men's alcohol consumption and DVA have been consistently evidenced as a widespread problem (e.g., Leonard & Quigley, 2017). Some researchers have further observed that intoxication may explicitly or implicitly mitigate perpetrator agency in DVA (Leonard, 2005). For example, Wood (2004) found that perpetrators justified and sought to disassociate from their violent behavior by attributing it to intoxication. Relatedly, female victims of DVA and other forms of gender-based violence constructed themselves as responsible for their partner's violence (e.g., Towns & Adams, 2016). Women may be met with stereotypical attitudes (e.g., victim-blaming, rape myths) from institutional parties, including the police, judges, and magistrates, at each stage of the criminal justice system (e.g., Fugate et al., 2005). Because attitudes about women's culpability in their own victimization include their level of intoxication (e.g., Finch & Munro, 2005), women may preemptively account for drug and alcohol consumption when reporting offences to the police (MacLeod, 2016).

Despite its high prevalence, formal reporting of DVA that initiates legal proceedings is disproportionately low. Systematic reviews of what constitutes barriers to help-seeking for gender-based violence (e.g., Lelaurain et al., 2017) include a fear of being disbelieved and of retribution from perpetrators. Furthermore, when victims *do* overcome barriers, and make formal complaints to the police, they report negative experiences (Lelaurain et al., 2017; Stewart et al., 2023). And so, in addition to asking victims about their experiences of reporting, the opportunity to systematically analyse live reports made to the police can help us better understand and determine what constitutes these poor experiences.

### Seeking Help for Domestic Violence

Our article is situated within a large body of conversation analytic research on help-seeking in both helpline and emergency service contexts. Perhaps the best-known work on the latter is that of Whalen et al. (e.g., Whalen et al., 1988; Whalen & Zimmerman, 1987; Zimmerman, 1992), who described the “achieved organization” of calls to U.S.-based 911 services and their composite sequences, from opening/identification; complaint/request, the “interrogative series” of questions and answers; response/promise of assistance, and closing (Zimmerman, 1984). Kevoe-Feldman and Pomerantz (2018) indicate that the promise of assistance is crucial in that it “serves to mark the end of the sequence that was initiated with a request for assistance or service, while simultaneously functioning as a preliminary move toward call closing” (p. 491). Relatedly, Kent and Antaki (2020) found that, in police emergency calls, the first substantive question asked by the call-taker to the caller reflected their judgment of how serious, relevant, and legitimate a request for help was, and was predictive of the outcome of the encounter. Furthermore, if the first question asked targeted a caller's address or location, it was strongly “dispatch implicative” (p. 644).

When it comes to domestic violence as a subcategory of reasons for calling emergency services, we know less than for other kinds of emergencies, though we know more about nonurgent requests to DVA charities and helplines, particularly through a series of studies conducted on such organizations in New Zealand (e.g., Tennent, 2019; Weatherall & Tennent, 2021). In one study, which, like the current article, focuses on how call-takers ask particular kinds of questions, Tennant and Weatherall showed that asking callers for their address could create tensions if the caller had left home to escape violence (Weatherall & Tennent, 2021). This shows that asking victims questions of any kind—even those that are seemingly routine and required by the organization—should be designed carefully for recipients. For instance, Flinkfeldt et al. (2022) showed that asking callers for their email address—another “routine” question—was a task done differently by call-takers in ways that either built in (e.g., “and what's your email?”) or removed (e.g., “do you have access to email at all?”) a presumption that callers had access or to knew how to use email. In this way, call-takers attended to the core interactional contingencies of recipient design—orienting “to the recipients, in whatever ways are relevant for the matter at hand, in producing their talk and actions” (Pomerantz & Heritage, 2013, p. 211) and preference organization—“how people systematically design their actions to either promote or undermine social solidarity” (Pillet-Shore, 2017, p. 1), and thus, overall, the progressivity of the interaction.

In the current article, uniquely positioned via our dataset of emergency calls to the police, we focus our analytic attention on the design of call-takers’ questions to callers—including victims of DVA—about alcohol and its consumption (e.g., “has anyone been drinking?”). These questions may appear to be something required by the institution and “routine.” Yet, like questions about a caller's address mentioned above, how questions are designed; where they are sequentially positioned, and so on, have interactional consequences. In focusing on live calls, we extend and deepen the insights made by other scholars about the negative experiences reported (post hoc to researchers, in, say interview studies) by victims of DVA. That is, we reveal the interactional practices that may *constitute* such experiences by examining help-seeking encounters as they unfold for participants in real time.

## Data and Method

In the United Kingdom, members of the public can report and seek assistance for DVA in two main ways: in an emergency, they can call 999 from a landline or mobile telephone; for less urgent reports, they can call 101. Calling 999 connects callers to a national call center where operators on a recorded line establish the need for police assistance (the caller is asked to select a specific emergency service from police, fire, or ambulance) and connects the call to a local call center. Calls made to 101 are connected directly to a local police call center. Call-takers elicit information from callers about what has happened, where they are, and who is involved, and then categorize them by severity, all recorded on a call log. This information is then passed to a dispatch team who reviews the information and allocates resources—including deciding whether to dispatch police assistance and how quickly.

As part of a research and training collaboration, we were granted access by a U.K. police service to the digital recordings of all incoming and outgoing calls made as part of the routine work of the organization (i.e., they were not recorded for research purposes). We collected a corpus of 192 calls to 999 and 101 made by members of the public reporting being victims to or witnesses of DVA prior to (in 2019) and during the first COVID-19 “lockdown” in England (in 2020). We know the restrictions designed to keep people “safe” resulted in those experiencing violence being isolated in their homes with the perpetrators of domestic violence (e.g., Bouillon-Minois et al., 2020). To identify calls to include in our collection, we used the U.K.-based definition of DVA as stated in Sections 1 and 2 of the Domestic Abuse Act 2021.

The Act considers the behavior of a person (“A”) toward another person (“B”) as domestic abuse if both person A and B are 16 years of age or over, are “personally connected; to each other and the behavior is abusive. The Act provides clarification that abusive behavior includes physical, emotional, economic, and sexual abuse and controlling and coercive behavior; “personally connected” refers to intimate partners, ex-partners, family members or individuals who share parental responsibility for a child.

The dataset did not include access to call logs, dispatch categorizations, or other metadata. We did not have access to training materials or “scripts” that the police force may (claim to) use. We also knew little about callers themselves since, for a variety of reasons, they often avoided disclosing their identity. Furthermore, callers often stayed silent on the telephone and communicated requests via nonlexical practices. This means that potentially salient variables such as gender identity, age, ethnicity, race, and sexuality, are perceptually (or designedly) unavailable or difficult to discern (e.g., a female-sounding voice may not correspond to the gender of the caller; callers may make incorrect assumptions about one party's age or ethnicity, see Flinkfeldt et al., 2022; Stokoe, 2015).

The data were analyzed using conversation analysis (CA). CA focuses on the systematic identification of composite actions in encounters, such as requesting, offering, or questioning, and their design and organizational structure. Conversation analysts usually work with recorded data, since they serve “as a control on the limitations and fallibilities of intuition and recollection” and enable “*repeated* and *detailed* examination of particular events in interaction and hence greatly enhances the range and precision of the observations that can be made” (Heritage & Atkinson, 1984, p.4, emphasis in original). Data were transcribed using Jefferson's (2004) conventions to enable analysis of the full range of practices that speakers use to take turns and build actions in interaction, including the pauses, gaps, speech perturbations, spontaneous talk, pace, and nonlexical vocalizations (and, relevant to the delicate nature of reporting and responding to reports of DVA, the sounds associated with upset, such as crying: Hepburn, 2004).

Although our dataset affords some kinds of comparative analysis between the 101 and 999 call types, as well as between the pre- and during COVID-19 lockdowns, this article focuses on the role played by alcohol across all data. Data processing agreements were drawn up by the police with the authors as research partners. All personal information was redacted on-site at the police station, the recordings anonymized and the project received ethical clearance. In the data presented, pseudonyms are used throughout for names and locations. Extract headings contain information about whether the call was made prior to or during lockdown, and whether the call was made to 999 or 101 (e.g., “Pri-75–999” refers to the 75th call to 999 in the prepandemic dataset). To investigate the extent of the relationship between DVA, alcohol, and help-seeking, we examined the data for all instances where this manifested in these initial reports. We found that alcohol consumption was topicalized by either the caller or the call taker in 17% of the calls. We chose to focus our analysis on questions formulated by call-takers and found 11 such instances, so in 5% of the corpus. Although we do not know if such questions are part of a “script,” we observed that they were not asked of every caller. We then focused on the design of call-takers’ questions about alleged victims’ and perpetrators’ alcohol (and sometimes drug) consumption, their action orientation, sequential environment, and interactional and institutional consequences.

## Analysis

Across the dataset, police call-takers typically asked a series of information-gathering questions to establish whether other emergency (e.g., ambulance, armed police) or nonemergency (e.g., social services) services may be necessary, and to tell attending officers what to expect on arrival. These included questions about injuries; whether children are present at the property; whether weapons are involved; parties’ alcohol (and drug) consumption, and, in the dataset collected during the first COVID-19 “lockdown” in England, about symptoms. We first examine questions about alcohol which typically appear after the call-taker has conveyed, explicitly or implicitly, that help has been, or is being, dispatched. In such cases, questions about alcohol are located in the environment of other information-gathering questions, and typically focus on the perpetrator's drinking. In the second section, we explore other sequential positions in which questions about alcohol appear, targeting both victim and perpetrator, and the potential implications for dispatch and overall experience of calling the police.

### Procedural Questions About Alcohol After Dispatching Police Assistance

Extract 1, split into parts a, b, and c, is from a single call. In this opening case, we show how calls about DVA progressed from the opening through to dispatching assistance (and “dispatch-implicative” actions) and subsequently, question(s) about alcohol consumption. The case exemplifies some of the core sequences that comprise our overall dataset, largely echoing findings from the research described above (e.g., in Kent & Antaki, 2020; Zimmerman, 1992). Extract 1(a) starts at the beginning of a 999 call made by a female caller reporting her partner's violence. Between lines 01 and 05, the national operator (OPR) connects the call to the local call-taker (CTR) who then addresses the caller (CLR) on lines 06–07.

**Extract 1.**
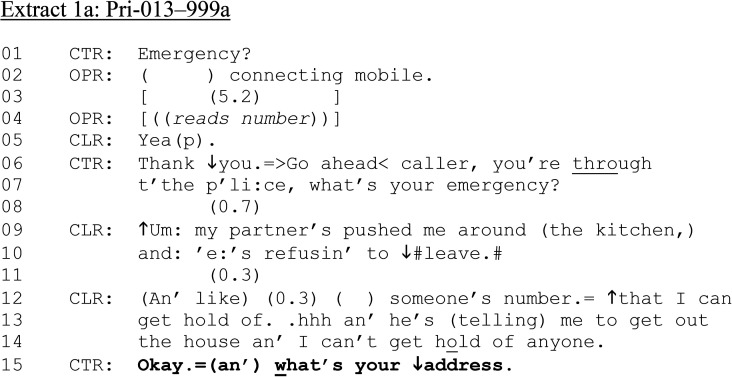
(a) Pri-013–999a.

In response to CTR's initial question, “What's your emergency” (line 07), CLR does not produce a direct, “high entitlement” request of the kind we also observe in our dataset (e.g., “I need yuh to come to 101 The Ridge”; Curl & Drew, 2008; Stokoe & Richardson, 2023). Rather, CLR formulates the circumstances she is currently experiencing (lines 09–14) in the “reason for calling” slot (see Hofstetter & Stokoe, 2015). Nevertheless, CTR treats this description as a request for assistance by producing a sequence-closing third response (“okay”) before launching a second turn-constructional unit to initiate the next sequence with the “dispatch-implicative” question, “What's your address” (line 15). At this point, CTR does not explicitly state that assistance has been dispatched. However, as Kent and Antaki (2020, p.644) note, “[t]he call-taker cannot send help if they do not know where to send it to. By implication, if the call-taker asks for the caller's location, this is at least consistent with (and is pragmatically taken to be) their intention to act on it and send the required help.”

The call continues in Extract 1(b).

**Extract 1.**
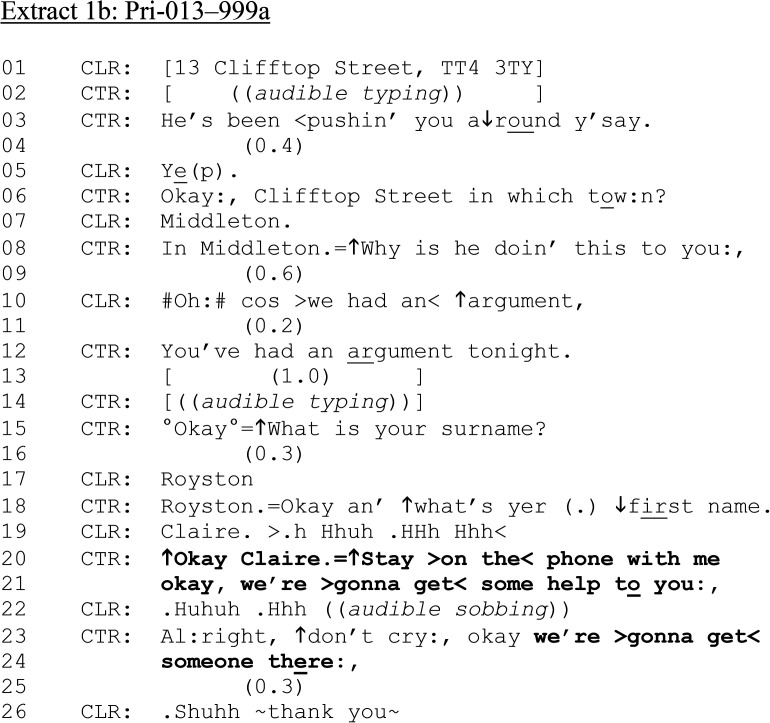
(b) Pri-013–999a.

Following and interspersed with eliciting address details (lines 01–08), CTR asks further questions about the incident (line 08) and CTR's name (lines 15 and 18). Note the explicit instruction for CTR to “↑Stay > on the < phone with me” prior to confirming “we’re > gonna get < some help to you:,” (lines 20–21). In cases where a caller is reporting an ongoing situation, and both parties are either assumed or confirmed to be in the same property, call-takers (attempt to) stay on the phone with callers until the police arrive. During this time, call-takers may request further details about the incident which can be reported to dispatched officers. But, as Kent and Antaki (2020, p.645, emphasis added) explain, “[t]here is an important caveat to note here about the dispatch-implicative nature of location solicits (whenever they occur in the call); namely, that they can be problematic when a caller then prematurely terminates the call, believing their request has been granted … [which is] *why police calls tend to have extensive insertion sequences between request and response*.”

Over the ensuing minute and a half of talk (not shown here), CTR asks a series of procedural questions about the incident (what has happened, whether CLR is hurt—potentially relevant for ambulance/medical services, the partner's details). We rejoin the call in Extract 1(c) as CTR reminds CLR to “stay on the phone.”

**Extract 1.**
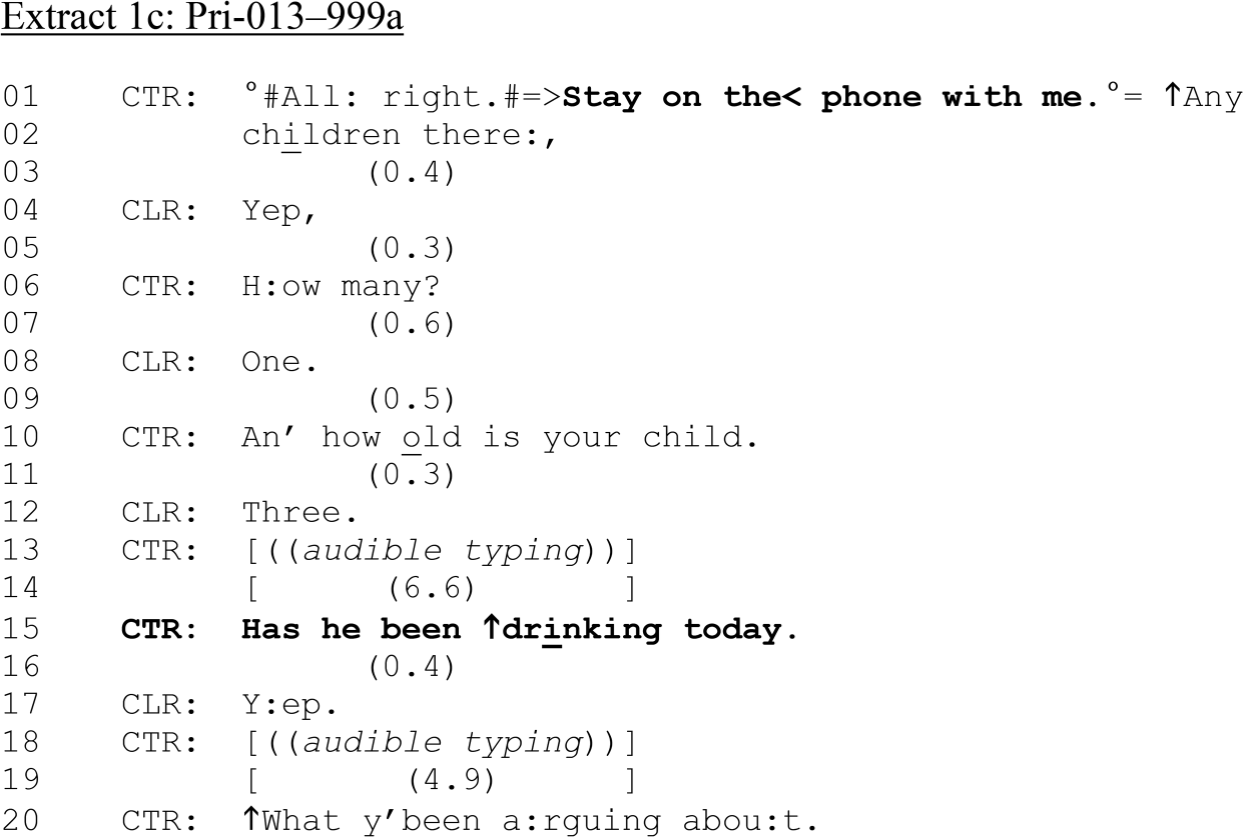
(c) Pri-013–999a.

Extract 1(c) illustrates how an alcohol consumption question, while not necessarily contingently formulated in response to prior turns-at-talk, may be produced following queries around the safeguarding of children and others (lines 01–12). At line 15, CTR asks, “Has he been ↑drinking today.” The “he” understood to be referencing the perpetrator of the violence CLR is reporting. The alcohol question, a yes/no interrogative (Raymond, 2003), is delivered in isolation from the surrounding talk; that is, periods of typing both precede and follow it. Although the question design does not necessarily push for a “no problem” response (e.g., “and any drinking?,” Heritage, 2005), and receives a slightly delayed “problem” response (“yep,” line 17), there is no further expansion. Later, CTR does return to the alleged perpetrator's alcohol consumption, during a 16-min conversation with several new topics, including CLR's child, the argument, and her mental health. Throughout this stretch of talk, CTR reminds CLR to stay on the line (as in line 01) and that help is on the way (as in lines 23–24 Extract 1(b)). Thus, the purpose of the questions seems at least as oriented to keeping the caller on the phone as eliciting dispatch-relevant information.

In Extract 2(a) and (b), a male victim has called nonemergency 101 to report his female ex-partner for entering and refusing to leave his home, something he assesses as “a li’l bit difficult” to say (lines 08–09). Although the 999 service is ostensibly for acute dispatch-relevant problems, and 101 may be resolved without dispatch—or indeed, within the call itself (Kent & Antaki, 2020)—in Extract 2, assistance is dispatched. Yet, the *male* victim has categorized his problem as a matter for 101. While we cannot be definitive, this supports research on the gendered barriers to disclosing and reporting (e.g., Hine et al., 2022; see also the CLR's account for calling at lines 08–09: “it's: a li’l bit difficult for me to ↑say this”). We join the conversation as CTR takes over from OPR.

**Extract 2.**
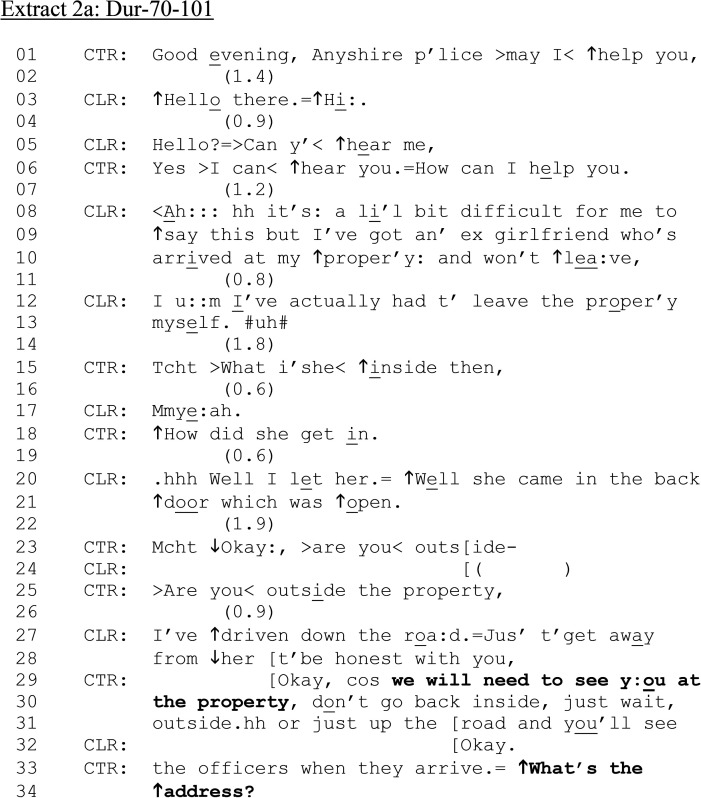
(a) Dur-70–101.

Unlike Extract 1, CTR asks “Anyshire p’lice > may I < ↑help you,” (line 01) rather than “what's your emergency,” relevant to the nonemergency purpose of service. However, like Extract 1, CLR describes an ongoing situation to formulate the reason for calling, which is followed by a series of questions from CTR to ascertain what is happening—and, specifically, the current location of both parties. At the end of Extract 2(a), CTR requests CLR's address, having also conveyed that the police are coming “cos we will need to see you at the property,” (lines 29–30) and also instructs him to wait outside and not return inside the house. Like Extract 1, it is after the confirmation of dispatch that the alcohol question is formulated.

**Extract 2.**
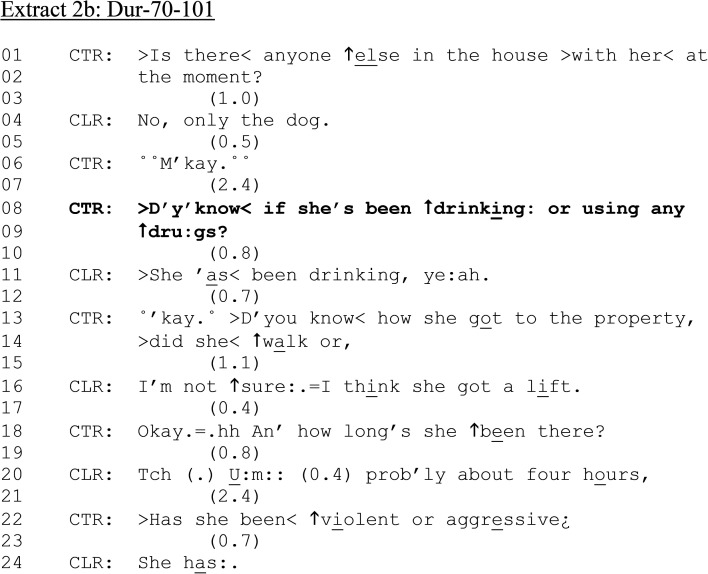
(b) Dur-70–101.

After confirming that his ex-partner is alone in CLR's house (lines 01–06), CTR formulates our target question: “Do you know if she's been ↑drink↑ing or using any↑drugs?” (lines 08–09). Rather than ask “*has she* been drinking or using any drugs,” which presupposes CTR will be able to confirm either way, the inclusion of a “do you know if” preface orients to the possibility that CLR may *not* know, as well as reducing his obligation to do so. Further, the question is negatively polarized (“any”) and thus grammatically tilted toward a negative response (Heritage et al., 2007). However, CLR confirms that his partner has been drinking (line 11) while deleting the “drugs” component of the question. Despite CLR's answer confirming his ex-partner's drinking, CTR moves on to a next question about how she “got to the property” (line 13), immediately adding a candidate answer (“>did she < ↑walk or,”). Driving may increase the “policeable trouble” already identified, and her culpability. However, CLR reports that he thinks his ex-partner “got a lift” (line 16). The issue of alcohol is not further expanded upon.

After establishing the length of time CLR's ex-partner has been at his house (lines 18–20), CTR asks if she has been violent or aggressive (line 22). The query is positioned after establishing whether the ex-partner had been drinking and can be seen as potentially linked in a series of procedural questioning. Yet, the sequencing of questions also orients interactants to the accountable behavior of the ex-partner. Alcohol consumption questions issued prior to establishing whether there has been domestic violence (and vice-versa) might have a bearing on how participants treat the reporting of these types of incidents. That said, in this example, there is a substantial gap before the issuing the domestic violence-relevant question (line 22), which is not “and-prefaced,” and thus might be understood as a “new topic” and disconnected from the alcohol question. Unlike Extract 1, CTR does not attempt to get CLR to stay on the line, likely due to CTR already being safely out of the house with a place to wait away from the perpetrator.

In the final case in this section, a hearably distressed female 999 caller is reporting that her partner has come to her flat and has “been hitting me in my face.” Extract 3(a) begins just after the caller has been connected by the operator.

**Extract 3.**
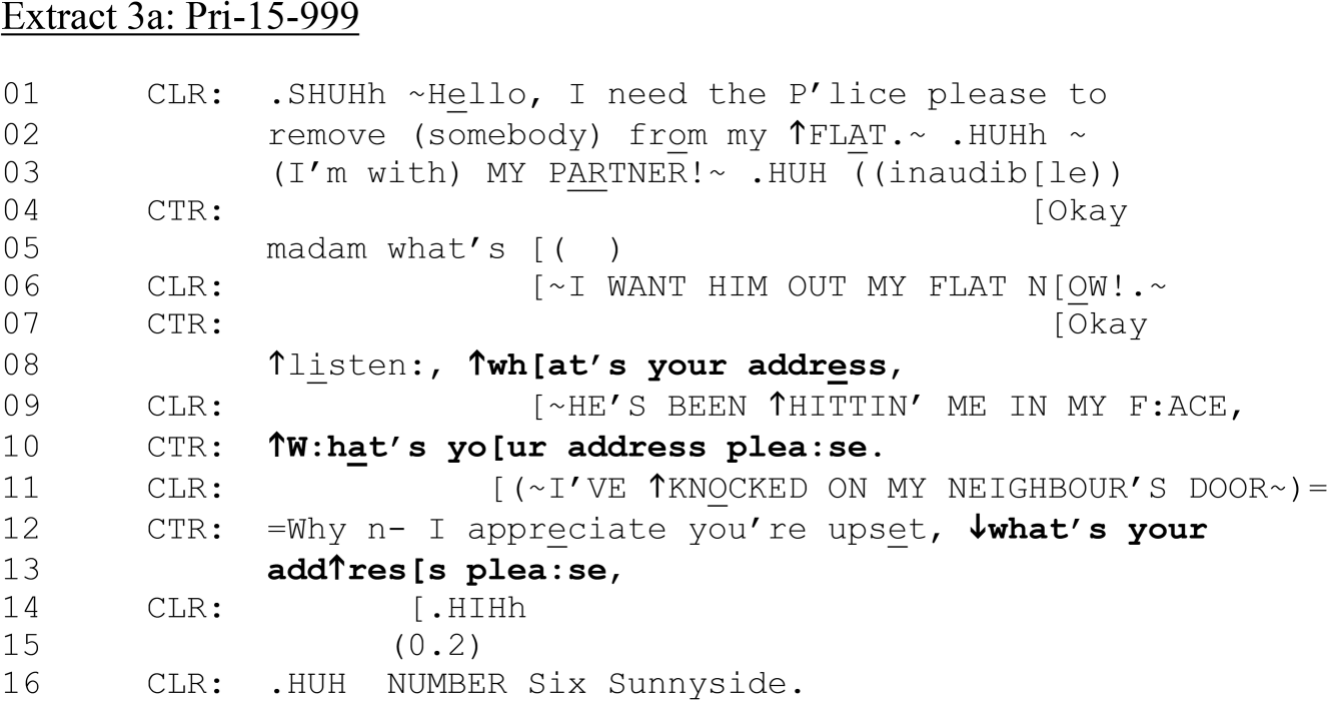
(a) Pri-15–999.

Unlike Extracts 1 and 2, CLR formulates a direct request (“I need the P’lice…”); like Extract 1, CTR elicits CLR's address, though after multiple attempts (lines 05, 08, 10, 12–13) and in overlap with CLR's continuing account of what is happening, at high volume. However, unlike previous calls, CTR does not confirm explicitly that help has been dispatched until later (see Extract 3(c)). After this extract, CTR asks questions about CLR's partner and whether she has lost consciousness. CTR has also requested that CLR to move to a place of safety. CLR has indicated that she attempted to seek safety at her neighbor's house but was refused entry due to the time of night (line 11 and on other occasions, not shown here). We rejoin the call in Extract 3(b) as CLR alerts CTR that her mobile phone battery has run out, further trapping her at her home.

**Extract 3.**
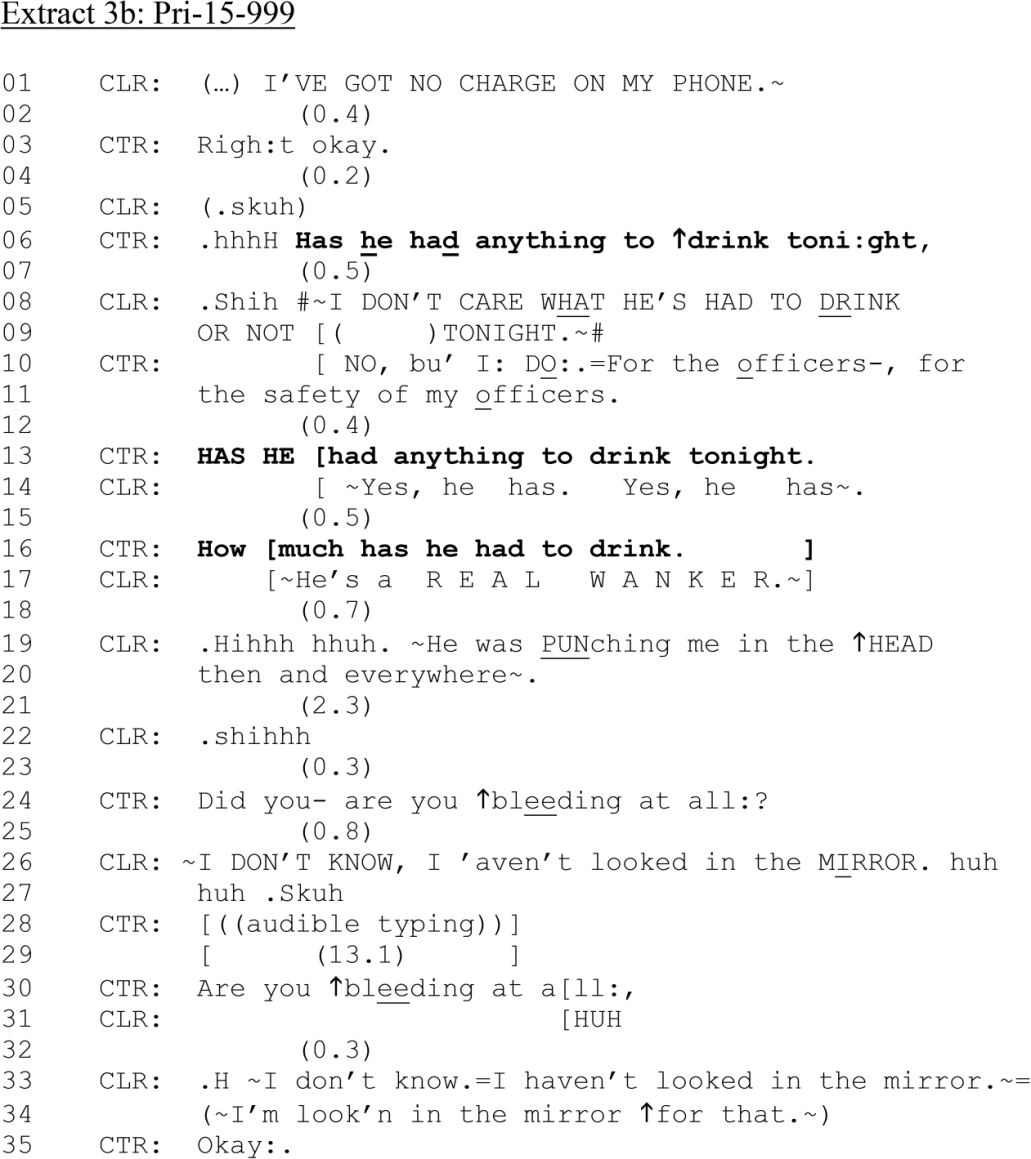
(b): Pri-15–999.

Like Extract 1, in which CLR is calling about her current partner (in contrast to Extract 2's ex-partner), CTR's question, “has he had anything to drink” presupposes that CLR has access to this information. However, rather than confirm or deny, CLR challenges the appositeness of any potential drinking to the matter in hand: “I DON’T CARE WHAT HE'S HAD TO DRINK” (line 08). Unlike other extracts, CLR's response topicalizes the issue of alcohol. CTR responds that she does care about his drinking, providing a procedural account “for the safety of my officers” (lines 10–11). This addresses the more problematic possibility, perhaps oriented to by CLR, that alcohol should not mitigate his behavior (Wood, 2004). CTR pursues an answer to the question at line 13, with raised volume on “HAS HE.” CLR responds in overlap hearing the trajectory of the question and repeats “Yes he has” twice (line 14). A further question at line 16 regarding amount of alcohol is not responded to as CLR pursues her own telling of the incident in overlap (line 17, 19–20).

In Extracts 3(a) and (b), then, there is misalignment between CLR and CTR regarding their respective agendas and the relevance of questions about alcohol. However, as with Extracts 1 and 2, CTR continues with questions relating to CLR's injury (lines 24–34). We rejoin the call again in Extract 3(c), which illustrates the live, high-stakes nature of the situation as CLR interacts with the perpetrator, which temporarily hinders communicating with CTR.

**Extract 3.**
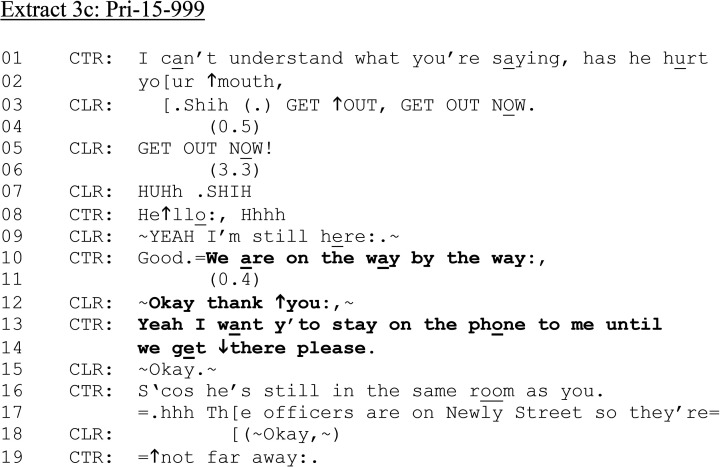
(c) Pri-15–999.

Only after a potential disconnection (lines 08–09) does CTR inform CLR that the police “are on the way” (line 10)—the addition of “by the way” exposing this information as new; something that CTR realizes they know but that CLR does not. CLR confirms that she has heard and appreciates that help is on the way (line 12). Again, this is followed with a directive to “stay on the phone to me until we get there please” (lines 13–14), with an account: “he's still in the same room as you” (line 16).

This section has shown that, and how, in calls to the police about DVA, call-takers ask questions about the alleged perpetrator's alcohol consumption (“Has he been drinking today”; “Do you know if she's been ↑drink↑ing”; “Has he had anything to drink toni:ght.”) *after* assistance has been dispatched. Decisions to dispatch are not impacted by responses to alcohol questions, which are asked procedurally, to gather information about the scene, perhaps to pass onto attending officers but also to keep callers on the phone. As Extract 3 shows, however, where help *is* on the way but has not been clearly communicated, the caller treated the question as objectionable and irrelevant to her case for assistance.

### Questions About Alcohol Including the Alleged Victim's Drinking Behavior

We now examine instances which again target the perpetrators’ alcohol consumption but are produced in different sequential positions as those analyzed in the first section. We also examine questions about callers’ own drinking behavior. We begin by returning to the call in Extract 1, in which we saw that the dispatch of assistance occurred early and prior to a series of procedural questions. We rejoin the call after a series of questions about the argument CLR reported, about their family dog (Extract 4).

**Extract 4.**
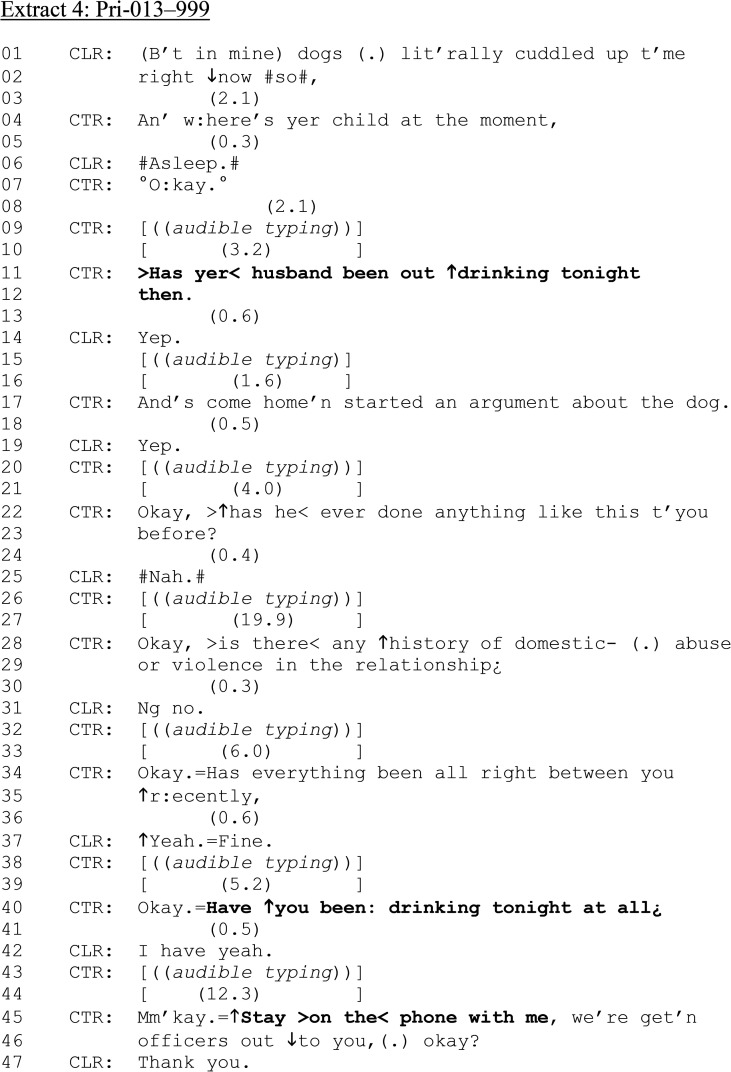
Pri-013–999.

After the sequence about CLR's argument with her partner (lines 01–03), CTR formulates an “and-prefaced” question “An’ w:here's yer child at the moment,” (line 04). There are multiple instances of gaps filled by CTR's audible typing through this extract, underlining the question's procedural-institutional function. At line 11, CTR issues the first of two alcohol questions: “Has your husband been out drinking tonight then.” Note, the turn-final use of “then,” which, coupled with the previous query regarding CLR's child, are potentially related to the reporting and managing of DVA and not as something directly relevant to safeguarding of either CLR's children or the soon-attending police officers. Further, and in contrast to previous examples, “*out* drinking” is hearable as sociable drinking. CTR's concerns as to whether the husband's behavior is commonplace (lines 22–23), the extent to which domestic violence is prevalent in the relationship (lines 28–29), and its recency (lines 34–35) are designed for “no problem” answers that also receive sequence-closing third confirmations from CTR (e.g., lines 07, 22, 28, 34, 40, 45).

The second alcohol consumption question is directed to CLR: “Have ↑you been: drinking tonight at all¿” (line 40). From Extract 1, we know that police have been dispatched, so responses to questions in the sequence above are not relevant to the CTR's decision to do so. As such, the caller-directed question may not carry the same moral weight as if issued predispatch (for instance, that victims may feel responsible for, or complicit in, DVA), which may be accounted for in the unproblematic design of CLR's response “I have yeah.” (line 42). Another typing-filled gap follows, but this is now a familiar pattern in the call and the next thing that happens is not expansion on the CLR's response but confirming dispatch (lines 45–46).

In contrast, Extract 5(a) and (b) is from a female caller to 999 who is reporting that her partner has hit her. She is hearably distressed, and her partner is in the vicinity of the call.

**Extract 5.**
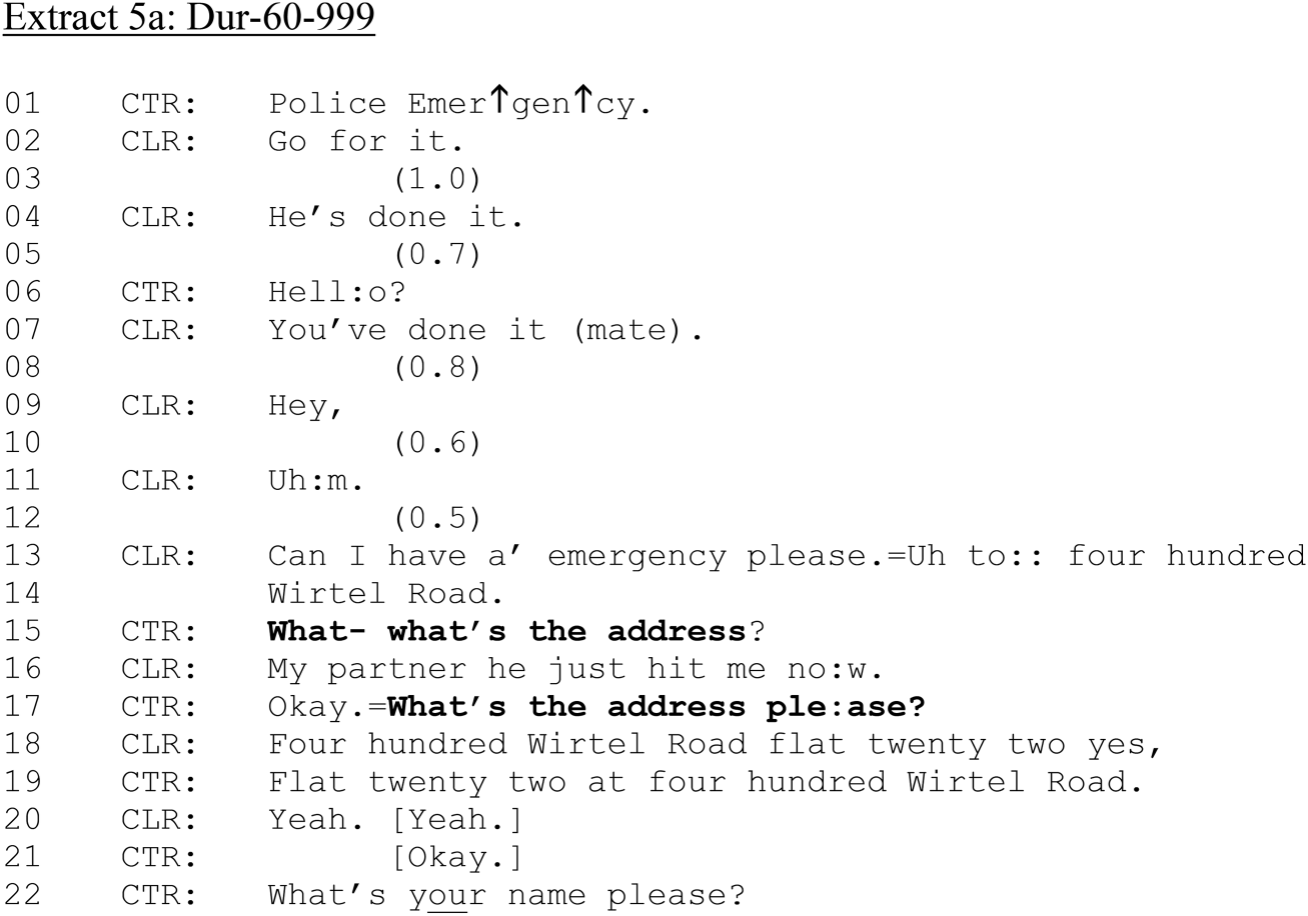
(a) Dur-60–999.

**Extract 5.**
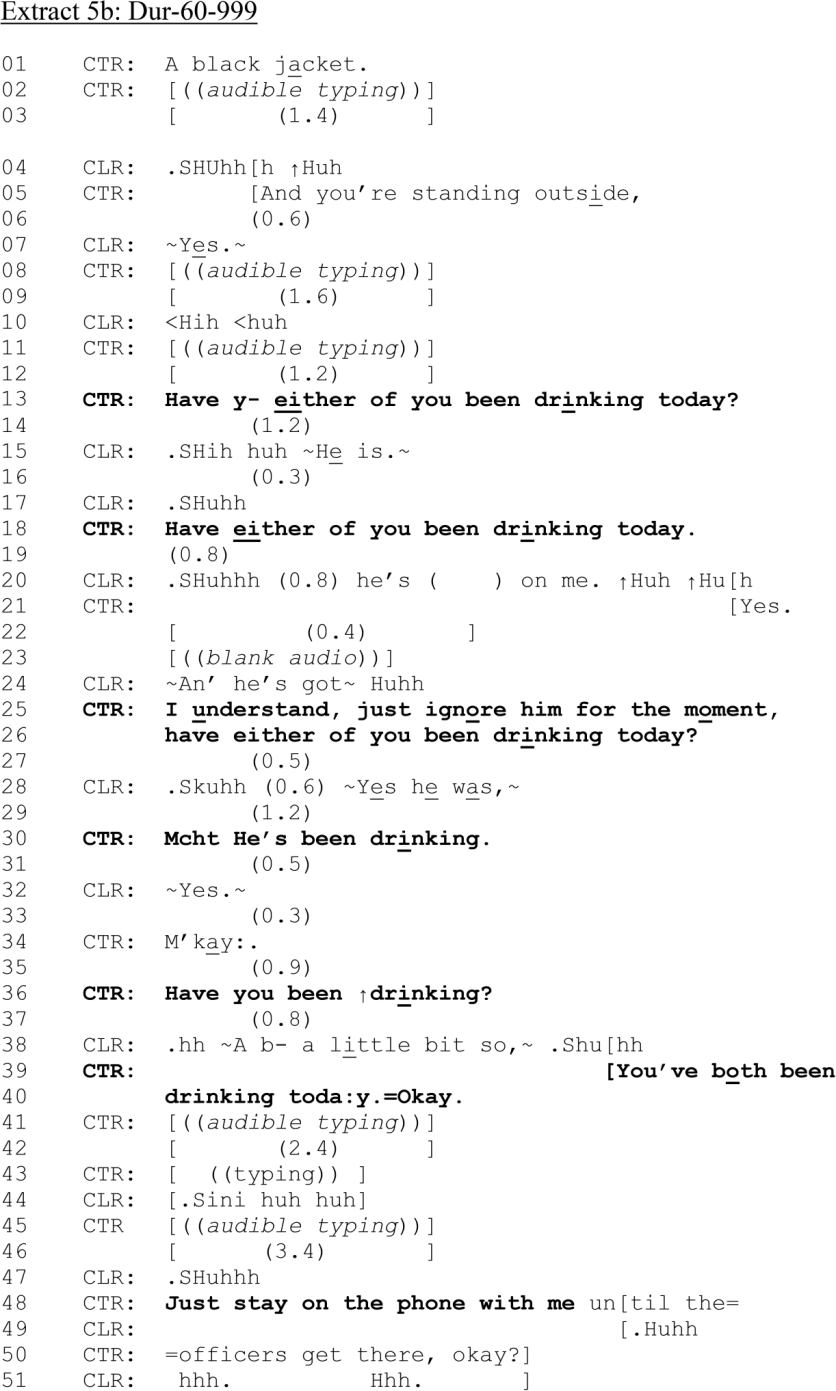
(b) Dur-60–999.

CLR is talking to someone else while CTR attempts to engage them (lines 01–07). At lines 13–14, CLR requests assistance and provides her address. CTR requests the address twice more (lines 15, 17), indicating trouble in hearing or understanding. CLR provides her address again at line 18 which CLR repeats and ratifies (lines 19, 21). Although the location has been established, CTR does not confirm that help will be sent (cf. Kent & Antaki, 2020). CTR then initiates a series of information-gathering questions (not shown here for brevity, but including questions asking if CLR has children, and if there is a history of violence). Throughout, CLR and perpetrator can be heard arguing. We rejoin the call as CLR is describing her partner's clothing.

Extract 5(b) shows how vague or ambiguously formatted questions can have interactional consequences for members, leading to extended sequences in which call-takers try to establish whether parties to reported DVA have consumed alcohol. After establishing what the perpetrator is wearing and where CLR is in relation to the potential crime scene (lines 05–07), the initial alcohol consumption question is issued (line 13). CTR begins to ask CLR “Have y-” but immediately repairs to include both parties in her question: “either of you been drinking today?” CLR treats the question as directed only at her partner: “He is” (line 15). CTR repeats the question to again include both parties (line 18), which is again responded to only in terms of the partner (line 21 and 24: note CLR's continued account of her partner's behavior “∼An’ he's got∼”). CTR issues the question a further time (line 26), and CLR again confirms that her partner has been drinking (line 28: “∼Yes he was,∼”). After a confirmation sequence (line 30–34), CTR now addresses the question specifically to CLR: “Have you been ↑drinking? (line 36). CLR positively acknowledges, yet does not say “yes” or “I have” (cf. Extracts 2(b), 3(b), and 5) but provides a downgraded and minimized confirmation “hh ∼A b- a little bit so,∼” (line 38), which resists the yes/no preference format in contrast to her previous “yes” response regarding her partner's drinking. The repair halts the production of “a bit” to insert “little,” further minimizing any drinking on CLR's part.

CLR's resistance to confirming her own drinking in response to the pursuit of the information by CTR illustrates how alcohol consumption queries can be treated as accountable by victims of DVA. It also shows how, through both the design of the question and confirmation of the response (lines 39–40), victims’ and perpetrators’ alcohol consumption in relation to DVA can be constructed as equivalent—they have *both* been drinking.

**Extract 6.**
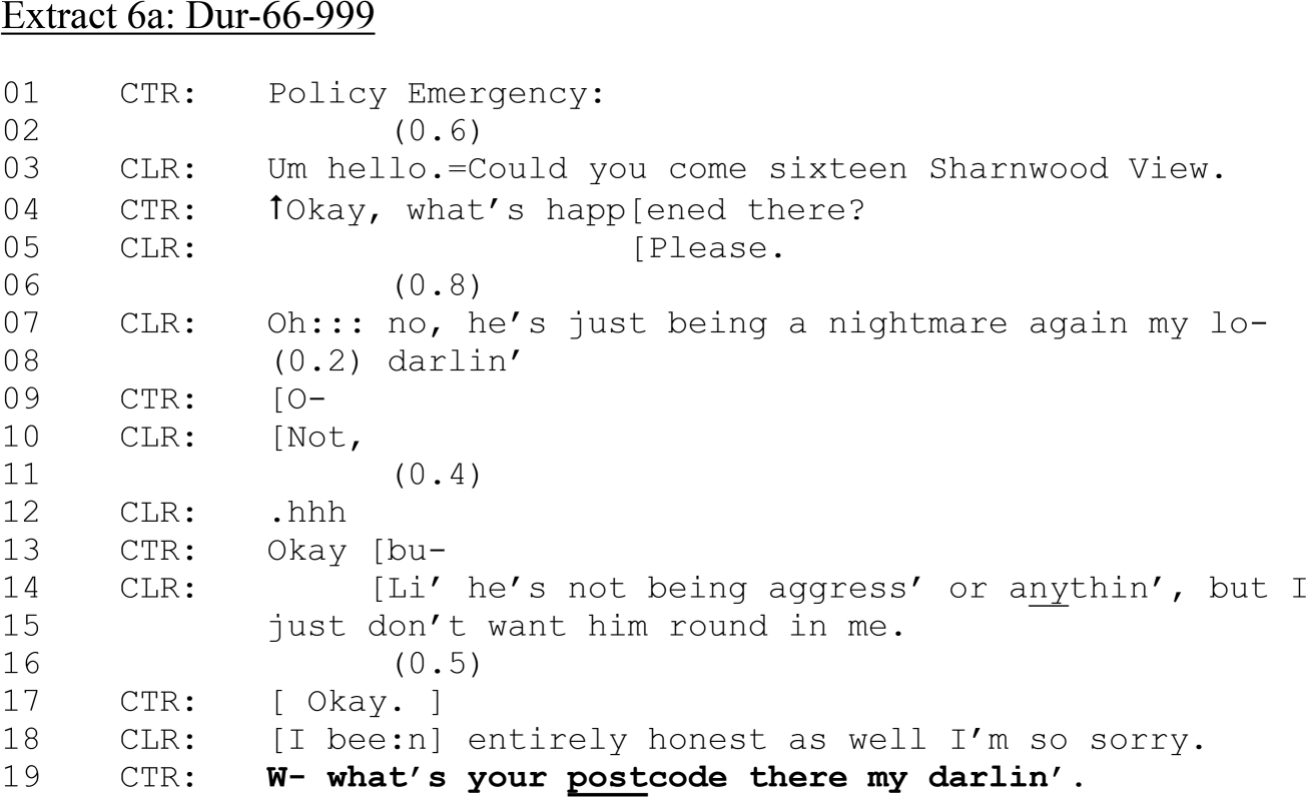
(a) Dur-66–999.

In Extract 6 (a) and (b), a female 999 caller has reported that a male is “being a nightmare” and refusing to leave her home.

As with Extract 5(a) (lines 13–14), CLR provides her address in her request for assistance. Yet the nature of the emergency is implied rather than made explicit (cf. Extract 5(a)). CTR thus pursues a reason for calling (line 04). CLR's account, “he's just being a nightmare again” implies a partner, but does not use an initial reference category shown in previous cases (partner, ex-girlfriend), but instead uses a “subsequent” reference in first position (Schegloff, 1996). This, together with the term of endearment CLR uses to CTR (“my lo- (0.2) darlin”), reduces the urgency of CLR's request by its “familiar” tone, along with the apparent change of state (line 07: “Oh::: no”) and the minimizing of his behavior (“just”). Nevertheless, CTR asks CLR a dispatch-relevant question (line 19), in so doing, enabling the police to pinpoint CLR's location.

As the call unfolds, CTR requests CLR's full address and establishes who the perpetrator is in relation to CLR and what exactly has happened. CLR reports that she has asked a man to leave, and he has refused. During this call, CLR's speech is hearably slurred; she repeats herself, and claims that COVID as has sent everyone “stir crazy.” The call continues:

**Extract 6.**
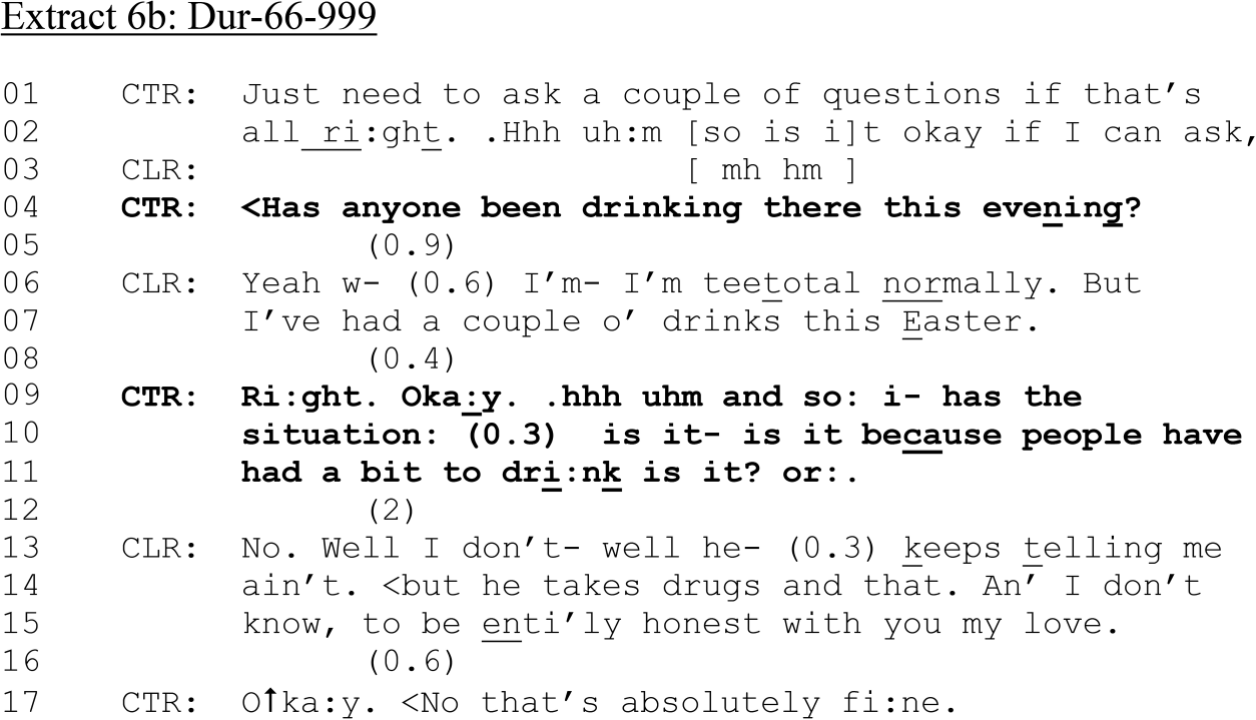
(b) Dur-66–999.

In Extract 6(b) we see the tentatively formulated (“Just need” and “If that's all right/is it ok if I can ask”) presequence (lines 01–03), and the alcohol question turn is ambiguously designed in terms of who it may be referring to—“has anyone been drinking…” (line 4). This stands in contrast to “has your husband…,” “have you…,” “has he…,” present in previous examples. In response, CLR produces a slightly delayed account in which she treats the question as directed at her, marking out her drinking as atypical “I’m teetotal normally” (line 06) but also that the occasion warranted alcohol consumption, having “had a couple o’ drinks this Easter” (line 07). In pursuing whether alcohol consumption had a bearing on her partner's behavior, CTR designs the referent in ambiguous terms: “is it because people have had a bit to dri:nk” (lines 10–11). CLR treats the question as directed toward her partner. It is also worth noting here, that despite a postcode being elicited from the caller, no explicit confirmation of dispatch has occurred.

**Extract 6.**
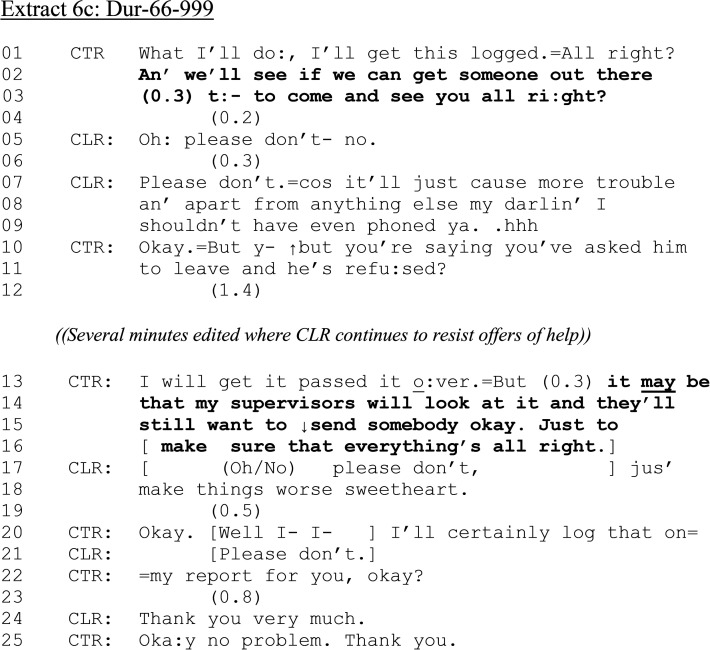
(c) Dur-66–999.

CLR exploits her response turn (i.e., the turn is through-produced in a way that effectively takes another turn delimiting response relevance), to announce her partner's drug taking—“but he takes drugs and that” (line 14). The turn itself is hearably disfluent and mitigated. The inclusion of “I don’t know” (lines 14–15) plus the honesty phrase “to be enti’ly honest with you” (line 15) conveys a dispreferred response as nevertheless sincere (Edwards & Fasulo, 2006). CTR confirms that CLR's lack of knowledge is “absolutely fi:ne.”

During the next 20 s, CLR confirms that there has been no violence but that she has just “had enough.” We rejoin the call as CTR informs CLR of the next steps in responding to her initial request to attend CLR's address.

Despite having asked for assistance at her address (Extract 6(a), line 03). CLR now rejects (line 05) and contests CTR's course of action to send officers “cos it’ll just cause more trouble” (lines 07–09) and “make things worse” (lines 17–18). While we cannot know for sure, her accounts orient to the “fear of retribution” barrier to reporting DVA. This may be why CTR does not accept CLR's requests not to come (including several minutes omitted for brevity), producing her own accounts for not letting the matter drop (“but you’re saying you’ve asked him to leave and he's refu:sed?”; “Just to make sure that everything's all right”), including invoking third parties who may “still want to send somebody over.” Whether call-takers should persist in the face of (perhaps anticipated) resistance from victims of DVA makes this call a particularly interesting case, since at the end CTR states that she will “certainly log that” in her report, referring to CLR's resistance, which does not necessarily alter the overall course of action.

In the final extract, a female 101 caller has been involved in an “altercation” at her ex-boyfriend's house which the police have attended and she is now back at her property. Interestingly, this means both of our 101 calls involve ex- rather than current partners. The caller reports that her phone remains at her ex's address.

**Extract 7.**
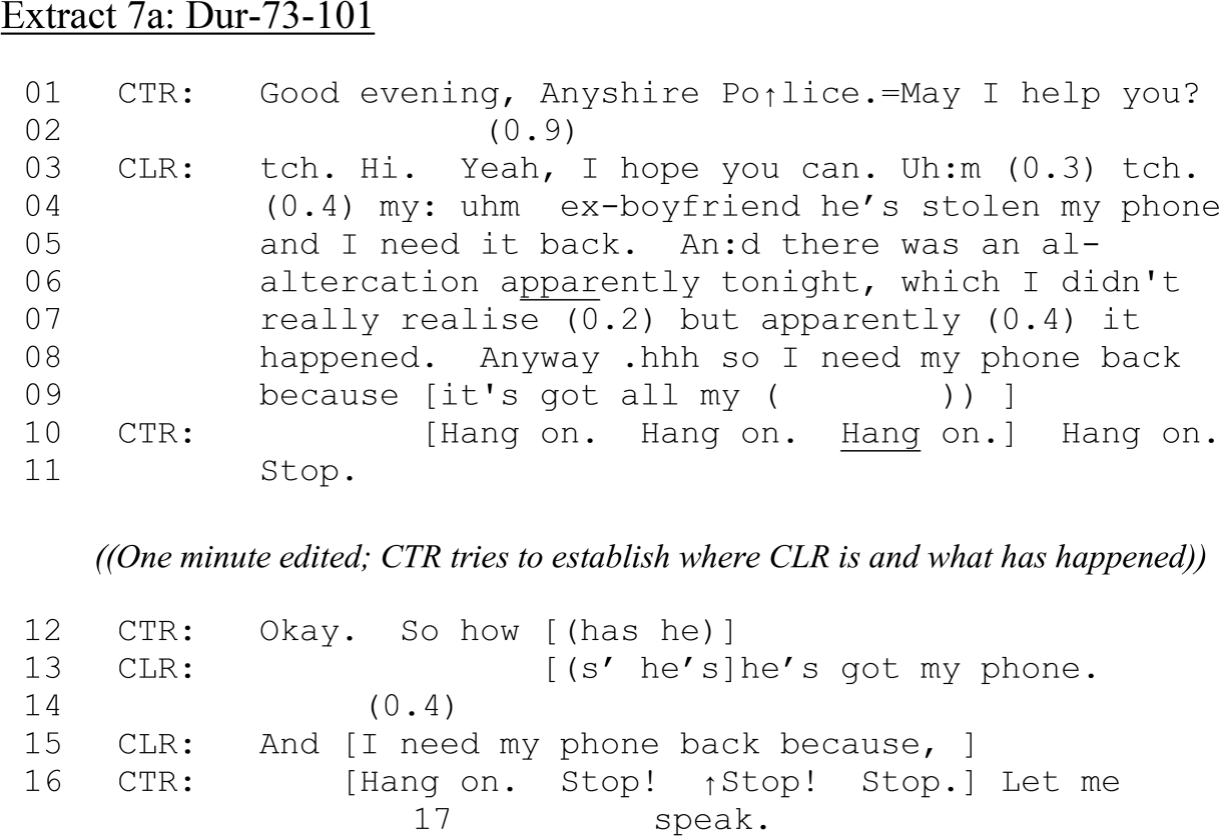
(a) Dur-73–101.

Throughout the call, CTR admonishes CLR for talking in overlap (e.g., lines 10–11, 16–17). Although CLR's partner's address has been established, CLR is not there, making possible dispatch decisions unclear. CLR reports that, after the “altercation,” her phone remains at her ex-boyfriend's address, held by him, as she had “uncompromising photos” of him. At no point does CTR advise CLR that assistance will be dispatched. However, we rejoin the call as the alcohol consumption question is issued.

**Extract 7.**
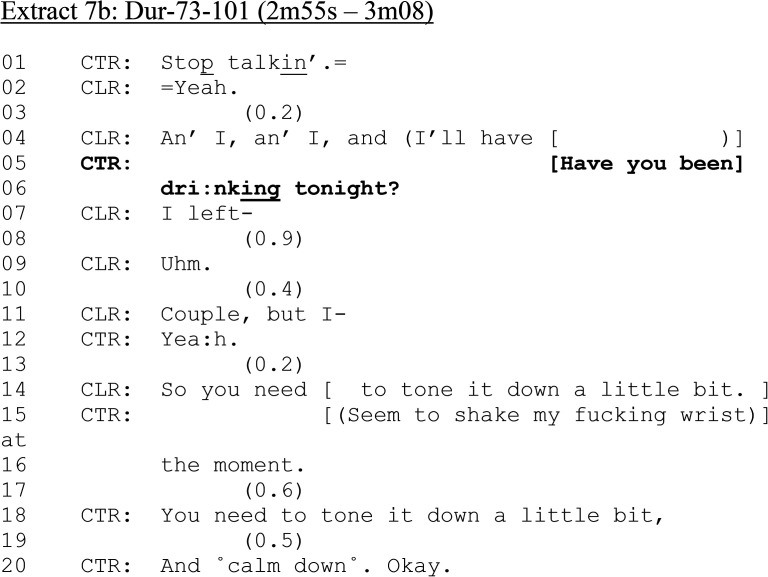
(b) Dur-73–101 (2 m 55 s–3 m 08).

At the start of the extract, CTR issues a directive to “stop talkin;” (line 01), which is initially met by CLR (line 02) but then she begins to talk again, somewhat incoherently (line 04). It is here that CTR, as an incursion into CLR's turn, asks her if she has been drinking tonight. The design and placement of the question makes it a vehicle for accounting for CLR's conduct, (talking incoherently and in overlap), taking on a moral, accusatory valence, rather than as one in a series of postdispatch procedural questions.

Immediately following CTR's question, CLR apparently continues with whatever she was saying earlier (“I left-”) but abandons this to address the question. After two gaps and hesitation (lines 08–10), CLR confirms (and, with possible moral valence, admits) she has been drinking with “couple.” Like Extract 5(b) (“∼A b- a little bit so,∼”), CLR does not provide a yes/no response to CTR's interrogative-formatted question but one that minimizes any drinking on her part. Furthermore, the relevance of any drinking is disclaimed with “but” (line 11). CTR responds at line 12 (“Yea:h.”) to confirm CLR's response, but her confirmation, along with the question itself, designed to halt the production of CLR's incoherent turn, hearable as an account for the way she is talking, and possible accusation, conveys a “question with a known answer” quality. CTR goes on to formulate the upshot of the alcohol question (“So you need to…”) to caution CLR to “tone it down a little bit” (line 14, 18) and “calm down” (line 20).

The sequence is an outlier in our corpus. However, it does demonstrate the extent to which alcohol questions can be vehicles for multiple actions. It also shows that alleged victims of DVA can and do face such actions. Extract 7 is resolved by CTR proposing she will “get the officers to contact you: and deal with this for you” although there is confusion around whether this actually happens.

## Discussion

This article has investigated the relationship between alcohol and DVA, and the (under)reporting of the latter, as they are made manifest in alleged victims’ live requests for assistance to the police. Our dataset, and particularly the incidence of police questions around alcohol consumption, have afforded us a unique opportunity to analyze their occurrence, location, action, and reception by callers—and provide an analysis that deepens what we know about DVA and victim-blaming, including the negative experiences callers report when disclosing DVA to the police.

Using CA, we found that questions about alcohol—and, specifically, the alcohol consumption of alleged perpetrators and/or victims—were broadly located in two sequential locations in the overall occasioned structure of the calls: first, after conveying to the caller that assistance was being or would be dispatched, and second, before dispatch was confirmed (or that a decision had clearly been articulated). In addition to the differing sequential locations, we also found differences in how the questions were formulated and responded to. In postdispatch sequences, call-takers largely asked questions about the alleged perpetrators’ drinking, not the callers. In these cases, questions were embedded in a series of other procedural questions which were sometimes seemingly designed to keep the caller on the phone while assistance was en route. Questions that targeted the callers’ drinking, often located before dispatch was confirmed, either explicitly (e.g., “have you been drinking?”) or indirectly (e.g., “has anyone been drinking there?”) initiated longer sequences and may impact dispatch—if not its occurrence, its immediacy.

A key distinction between types of questioning, including about the caller's drinking, was whether callers answered in ways that met the preference organizational constraints of the inquiry. For instance, yes/no interrogative questions about a perpetrator's drinking, postdispatch, generally received a fitted yes/no response (e.g., CTR: “Has he been drinking?,” CLR: “Yes”). This was typically followed by a sequence-closing third confirmation (e.g., “Okay”) from the call-taker and a next sequence that did not expand upon the caller's answer. In one instance, the caller challenged the relevance of her partner's drinking—perhaps not wanting to mitigate his violent behavior (Wood, 2004), though of course, we cannot know this for sure—and, in response, the call-taker provides a safeguarding account for asking on behalf of the attending officers. When yes/no questions were asked of callers’ own drinking, we found that expanded sequences followed, with a moral valence. Rather than responding with yes or no, callers’ responses minimized their own drinking and also disclaimed its relevance. We also found instances both of call-takers pursuing offers of assistance in the face of callers apparently withdrawing their complaint for fear of reprisals (relevant to understanding barriers to reporting) and of call-takers asking questions about alcohol to account for callers’ own behavior (relevant to victim credibility, fear of being believed and/or potentially culpable for the events in hand). Our qualitative analysis and small number of instances means we cannot make statistical claims. Nevertheless, our data enables us to show how these well-established issues in the DVA literature may manifest in actual calls.

In conclusion, our analysis has shown that information-seeking questions—even seemingly routine ones—can be asked in different ways that shape the subsequent trajectory and outcome of an encounter. We have seen *how* questions about alcohol consumption are asked of those seeking assistance from the police in the case of DVA, and *where* they appear in the overall landscape of the call, may contribute to feelings of blame or being disbelieved (see also Richardson et al., 2019). This may be especially so (a) if questions about alcohol are asked in ways that do not appear to be “routine” or “procedural”; (b) if questions initiate extended sequences of further conversation; and (c) if call-takers have not already confirmed that they will dispatch assistance to the caller. Understanding the consequences of the design and location of alcohol consumption questions can help the police in developing guidance and training for call-takers when there is a requirement to understand the levels of intoxication of the parties involved in the incident. By examining real calls in this way, and considering underreporting as an interactional problem, our findings strengthen and corroborate existing research findings on barriers to reporting DVA and, in turn, highlight the continued need to understand the lived—and live—experience of victims.
